# Adulteration of Saffron

**Published:** 1845-08

**Authors:** 


*Adulteration of Saffron.*—J. Muller recommends concentrated sulphuric acid as the most certain test for saffron, for it immediately turns the color of pure saffron to indigo blue, (it however soon changes to dark red and brown.) The leaves of *crocus vernus*, which form the most frequent adulteration, are colored of a dark green by sulphuric acid.—*Archiv. der Phar.* in *Bost. Med. & Surg. Jour.*

